# Cellular receptor binding and entry of human papillomavirus

**DOI:** 10.1186/1743-422X-7-2

**Published:** 2010-01-06

**Authors:** Tan Letian, Zhang Tianyu

**Affiliations:** 1 Department of Otolaryngology, Eye and ENT Hospital of Fudan University (formerly Shanghai Medical University), Shanghai, China; 2 Department of Otolaryngology, Eye and ENT Hospital, Fudan University, Shanghai, China

## Abstract

Human papillomaviruses (HPVs), recognized as the etiological agents for the skin, plantar, genital, and laryngopharyngeal wart, have been previously in numerous studies demonstrated to present a close link between HPV infection and certain human cancers, some putative candidates of HPV cell receptor and possible pathways of cell entry proposed. This review was to highlight the investigations and remaining questions regarding the binding and entry process.

## Introduction

As the well-recognized etiological agents for the skin, plantar, genital, and laryngopharyngeal wart, human papillomaviruses (HPVs) have been proven of a close link between HPV infection and certain human cancers. And considerable effort has been made in developing a prophylactic vaccine and devising effective treatments of HPV-induced lesions. It has been found that the early events of HPV infection such as cellular receptor binding and entry into susceptible cells could provide potential targets of inhibiting the spread of a HPV infection.

HPVs are nonenveloped double-strained DNA viruses about 55 nm in diameter with an approximately 8-kb genome in the nucleohistone core, and their capsids are composed of two virally encoded proteins, L1 and L2, with L1, the major capsid protein, mainly responsible for initial binding to the cell surface, and arranged in 72 pentamers which associate with T = 7 icosahedral symmetry [1]. Even in the absence of other viral proteins, L1 self-assembles into empty capsid or virus-like particles (VLPs) [2]; L2 is incorporated into VLPs when coexpressed with L1, i.e. L1/L2 VLPs, in insect or mammalian cells [1]. It is well known that difficulties in generating HPVs *in vitro *hinder the study on the pathway of infection. Recent research predominantly utilized synthetic HPV particles, such as VLPs, HPV-based gene transfer vectors known as pseudovirions (PsV), or papillomavirus genome-containing *quasivirions *(QV). VLPs, including L1 VLPs or L1/L2 VLPs, are widely used for the binding assay and vaccine production. PsV, produced for the studies on internalization, are composed of the VLPs packaging or attached to a reporter gene whose subsequent expression is used to identify and quantity pseudoinfected cells [3]; QV are generated as in the case of PsV in that the transfection of L1 and L2 codon-optimized expression plasmids, in addition to full-length, recircularized HPV genomes, into 293T or 293TT cells allows for efficient intracellular production of the native virion-like particles [4].

However, native virions are generated in stratified and differentiated epithelia and are thus synthesized only during a natural infection or in an organotypic culture [4]. For years, the low productivity of organotypic culture garnered too few virions for many low sensitivity analyses. Recently, Broker's group established a highly efficient and reproducible system that generated autonomous HPV-18 genome in the primary human keratinocytes, the organotypic raft cultures of which recapitulated a robust productive program, which suggested potential value for HPV genetic dissection and a faithful *ex vivo *model for investigating infections and interventions [5,6].

It is well known that HPVs, prior to a successfully established infection, have to experience a complicated process to bind to and enter the host cell. Our review was intended as an update on the cellular receptor and endocytic route of HPVs, with a focus on each putative receptor and a possible pathway based on the previous evidence derived from the literature review. In addition, we briefly clarified the function of L2 protein in HPV infection.

## 1. HPV receptor binding and virus-host cell interaction

The identification of HPVs cellular receptor began with the observation of interaction between papillomaviruses and cells. Roden et al observed that BPV-1 virions, as well as VLPs of HPV-16, were capable of binding specifically to several mammalian cell lines of fibroblastic and epithelial origin [1]. Afterwards, HPV-11, -16 and -33 VLPs were reported to have bound to or entered a wide range of cells [7,8]. These findings suggested that papillomavirus receptor was a widely expressed and evolutionally conserved surface receptor. Later, Qi et al reconfirmed the outcomes, and, furthermore, proved the receptor as a trypsin-sensitive structure and identified a B-cell line DG-75 that did not bind VLPs, which was critical to the investigations to come [9].

### 1.1 α6 integrin

The first candidate of cellular receptor was nominated as α6 integrin [2,10,11]. The integrins, heterodimeric glycoproteins comprising of α and β subunits, are expressed in a variety of cell types, primarily involved in cell-matrix and cell-cell interactions, and as the previous researches revealed, capable of acting as virus receptors for initial binding and/or internalization, as in the case of echovirus (α2β1), coxsackievirus (αvβ3), hantavirus (β3), adenovirus (αvβ3/5), and foot and mouth disease virus (αvβ3) [2].

At present, 17 types of α subunits and eight of β subunits are known, of which α6, β4, and β1 have been reported to be involved in HPV binding. And it has been clarified that α6 integrin played a leading role in this process with the evidence that a monoclonal antibody against the α6 integrin subunit reduced the binding degree of VLPs, while anti-β1 or anti-β4 antibodies did not [2]; that HPV6 bL1 VLPs failed to bind to DG-75 [2], whereas VLPs did to the genetically modified DG-75 expressing α6 integrin [10]; that the binding degree of HPV-16 VLPs to each cell type varied not with the expression level of β subunits, but with that of the α6 integrin [11]. In addition, the α6 integrin was known capable of invoking a transductive signal pathway to initiate DNA replication in keratinocytes, which could be an ideal condition for viral replication [10].

Although both the α6β4 and α6β1 heterodimer were capable of binding VLPs *in vitro*, only the former was supposed to function as a HPV receptor, which was concluded from the results that the α6 subunit associated preferentially with the β4 in epithelial cells [12], and the α6β4 complex was expressed exclusively in the basal cellular layer of the stratified squamous epithelium [13], which was presumably the only site of productive PV infection, while the α6β1 was found in relatively more cell types and sites unrelated to HPV infection, which might partly explain why HPVs were reported to be able to bind to a wide range of cells *in vitro *that were not the natural host of HPVs [10]. And the consistent results from Evander et al showed that the human laminin, the natural ligand for the α6β4 complex, was capable of blocking the binding of VLPs to HaCaT cells in a dose-dependent manner [2].

It is generally held that HPV infection is believed to occur as a result of exposure of basal cells to virus particles upon a minor trauma to the epithelium. During the wound healing, the α6β4 complex presented a high expression over the entire surface of the epithelial cells migrating to cover the focus [14]. Furthermore, the complex was constitutively endocytosed and recycled, with a rate of endocytosis of 1% to 2% of surface molecules per min, and with a recycle of the receptor facilitating the cellular migration, during which it moved in and out as the cell was advancing [15]. Therefore, a model of HPV infection was proposed that HPV particles bound to the α6β4 complex during the wound healing and were endocytosed to the microfilament network via the hemidesmosome [2], of which the complex is an integral part [16].

### 1.2 Heparan sulfate proteoglycans/Heparan sulfate

#### 1.2.1 Heparan sulfate proteoglycans/Heparan sulfate and HPV binding

Cell surface heparan sulfate proteoglycans (HSPGs), mainly syndecans and glypicans, are complex molecules composed of a core protein with covalently attached glycosaminoglycans chains, especially heparan sulfate. The glycosaminoglycans, comprised of alternating disaccharide units of uronic acid and amino sugars, are posttranslationally modified by sulfation and acetylation to various degrees, providing a variety of molecules with substantial sequence heterogeneity [17]. HSPGs are involved in a wide variety of biological phenomena, including organogenesis, angiogenesis, growth factors/cytokine actions, wound healing, and cell adhesion. Moreover, they are implicated as primary host cell receptors for many viruses, although most of them depend on secondary receptor proteins for efficient internalization [18].

As part of HSPGs, heparan sulfate was reported to play a critical role in the binding of HPVs to the cell surface: 1) the removal of heparan sulfate glycosaminoglycans on keratinocytes with heparinase or heparitinase resulted in an 80%-90% reduction of HPV-11 VLPs binding [19]; 2) the pseudoinfection of HPV-16 and -33 was inhibited by heparin, reduced with a decline in the level of surface sulfation, and abolished via a heparinase treatment [20]; 3) HPV-16, -18, -31, -33, -39, -45, -58, -59, and -68 VLPs possessed the ability to transfer genes into COS-7 cells in an efficient way, which, however, was inhibited when the pseudovirions were preincubated with heparin [21].

Of HSPGs, syndecan-1, instead of syndecan-4 and glypican-1, was reported to function as a HPV receptor. Evidence were: 1) when cells was treated with heparinase I, rather than with phosphoinositol-specific phospholipase C, which could remove most of the surface heparan sulfate, the degree both in their binding of VLPs and infection with HPV-33 pseudovirions was sharply reduced [22]; 2) K562 cell with ectopic expression of syndecan-1 could enhance its binding of HPV-16 VLPs, which otherwise possessed no HSPGs but minor amounts of molecules and thus weakly bound of VLPs [23]. Syndecan-1 was strongly upregulated during the wound healing, and widely expressed on the migrating and proliferating keratinocytes as well as on the adjacent hair follicles. Therefore, the basal keratinocytes, in addition to the suprabasal ones, when exposed upon a minor trauma or abrasion, overexpressed syndecan-1, thus upregulating strongly their ability to bind and internalize papillomaviruses *in vivo *[23].

#### 1.2.2 Conformation of HPV virions and HPV binding

The conformation of HPV particles is considered to be critical to the cellular binding. Initially, Joyce et al identified a conserved heparan-binding region on the carboxyl-terminal portion of HPV L1 protein through a sequence comparison of nine HPV types: HPV-11, -3, -13, -31, -58, -6b, -40, -7 and -42. This region was found to be located in the final 15 amino acid residues of the L1 protein of the general type XBBBBXB where B was Lys, Arg or His, which was similar to the XBBXBX and XBBBXXBX consensus sequences of the known heparin-binding proteins [19]. However, later studies reported that deletion of this region did not affect the interaction of HPV-33 VLPs with heparin [20], and the interaction was strictly dependent on an intact outer surface conformation of L1 [24], suggesting that the basic C-terminus of L1 was not sufficient for heparin binding. Moreover, the structure of the papillomavirus capsid, reported recently, showed that the C-terminus was not surface-exposed [25,26]. Taken together, these data indicated that the interaction between the capsid and heparin required an intact outer surface structure, which provided a conformational cluster of basic amino acids rather than a linear arrangement of positively charged amino acids [24].

Afterwards, Selinka et al, based on published and their own data, proposed that papillomavirus virions might exist in two conformational forms, the closed and open, the former the predominant species in solution, from which the binding of the surface receptors cause a transition in the virion to the latter, which might initiate internalization and uncoating [17]. Day et al later on demonstrated this model in details via a study on neutralization of HPV with monoclonal antibodies [27]. In the study, three anti-HPV-16 monoclonal antibodies were employed, which were H16.V5 (V5), H16. E70 (E70), and H16.U4 (U4). And the result showed that V5 and E70, recognizing overlapping epitopes present on the apex of the L1 capsomers [28], did not interfere with the virion binding to the cell surface, but neutralized infection by preventing the internalization of bound particles [27]; that U4, whose epitope was mapped to a C-terminus portion of L1, and was proposed to extend between adjacent capsomers [28], interfered with infection by preventing cellular binding, but did not interfere with the binding to ECM. These data suggested that interaction between HPV and cells was dependent on functional epitopes on the particle [27]. As in the case of the U4 epitope, it physically overlapped with a HSPGs binding site within cleft within which there might be a heparin-binding domain, which, however, might not be the site originally proposed by Joyce et al, and cell-induced conformational changes could expose the C-terminus of L1, resulting in a higher affinity binding between virions and cells. On the other hand, several epitopes, such as V5 and E70, once occupied, could prevent a necessary conformational change in capsid, or might induce a conformational change in another way, in order to block the binding [27].

#### 1.2.3 Structure of HSPGs and HPV binding

The structure of HSPGs is believed to be equally essential. For instance, O sulfation of HSPGs was sufficient for VLPs binding, which, however, was required together with N sulfation by pseudoviruses [17]. This, nevertheless, has received little research. So far, it has been recognized that 2-O-sulfate groups, primarily located on iduronic acid residues in heparin and heparan sulfate, glucosmine N-sulfate and in particular glucosamine 6-O-sulfate groups of the polysaccharide all contribute to the interaction with HPV-16 VLPs. In addition, eight monosaccharide units of heparin were sufficient for the binding of HPV-16 VLPs, which increased as the heparin chain prolonged in size from eight to 14 units, but decreased with 16 or more units [18].

It still remains mysterious whether α6 integrin or HSPGs is the genuine cellular receptor of HPV, for there are counterevidences and controversies for either. Several studies indicated that α6 integrin was dispensable for HPV-11 VLP binding to cells [19], for BPV-4 infection [29], as well as for HPV-16 and HPV-33 pseudoinfection [20]. On the other hand, HSPGs, especially heparan sulfate, was not required for HPV31b virions infection of human keratinocytes *in vitro *[3]. However, Johnson et al recently showed the opposite results using the murine cervicovaginal challenge model that *in vivo *HPV-31 infection was dependent on HSPGs [30]. We put forwarded at least three possible explanations to the discrepancies between the outcomes. In the first place, it was largely that the different assay systems were employed since a standardized one was not available, where the viral particles could be VLPs, pseudovirions, or authentic virions; and the cell lines, diversified, including those derived from malignant carcinomas, such as Hela and HaCaT, etc, and the normal keratinocytes from human beings or animals. Different kinds of viral particles required different concentrations in assay. For example, MOIs in the setting of authentic virions ranged from 5 to 50 viral genome equivalents per cell, but reached thousands to tens of thousands per cell in most cases of VLP binding or pseudovirion pseudoinfection [3]. And different cell lines presented distinct characteristics. Those transformed cells, which lost some of their epithelial characteristics, might result in disparities. Second, HPVs of different types employed distinct molecules as their own primary receptors. This could be the simplest explanation, which needs further evidence. In addition, further studies suggested that HPV infection was likely to engage more than one cellular surface protein, as in the case of a secondary receptor [10,19,20,23]. It was possible that HPVs utilized this strategy for infection that initial binding to a primary receptor and then transfer to a secondary receptor allowing for invasion of cells. Thus, it was most likely that both α6 integrin and HSPGs, functioning as primary or secondary receptor, contributed individually or in combination to the process. It should be noted that a virus receptor means a host surface component involved in binding and facilitating a viral infection. Therefore, we believe that both α6 integrin and HSPGs can be labeled HPV receptor, and that more receptors will be identified in the future.

## 2. HPVs to enter cells via distinct pathways

Intriguingly, most of the studies proved that different types of HPVs entered cells in distinct pathways, including clathrin-mediated endocytosis, caveolar endocytosis, and clathrin- and caveolae-independent endocytosis.

### 2.1 Clathrin-mediated endocytosis

**Figure 1:**
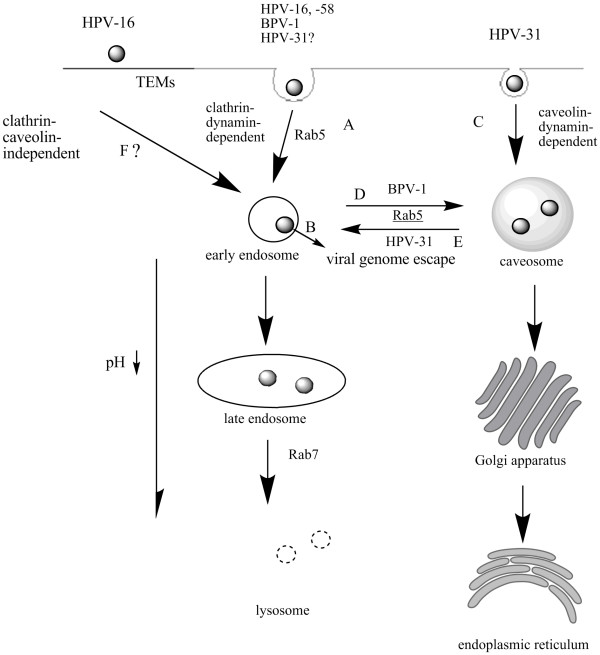
**Endocytic pathways of HPVs**. (A) HPV-16, -58, and BPV-1 entered via clathrin/dynamin dependent pathway. HPV-31 might enter via this pathway. (B) The decrease of pH led to endosomal escape of the viral genome. (C) HPV-31 entered via caveolin/dynamin dependent pathway. (D) and (E) Virions were transported between early endosome and caveosome. (F) HPV-16 might be internalized in clathrin- and caveolae-independent pathway, via TEMs.

It was found that clathrin-mediated endocytosis was the major cellular entry for many viruses. The binding of ligand to a specific receptor is widely recognized to result in the clustering of the ligand-receptor complexes in the coated pits on the plasma membrane (Fig. 1A), which then invaginated and pinched off from the plasma membrane to form intracellular clathrin-coated vesicles in progress to early endosomes in a Rab5- dependent manner before being fused with each other to form late endosome or lysosome, controlled by Rab7. And the molecules internalized via clathrin-mediated endocytosis experience a fast decline in pH from the neutral to a pH approximately 6 in the early endosomes transforming into the late endosomes and ultimately degrading in lysosomes, with a pH of approximately 5[31,32].

Day et al examined the pathway via which papillomaviruses infected cells using BPV virions and VLPs, and concluded that the pathway was accomplished via clathrin-dependent receptor-mediated endocytosis, and the viral capsid, unlike other molecules, presented a conformational change when the pH of the endosomal compartment decreased, resulting in the endosomal escape of the viral genome or genome/L2 complex (Fig. 1B) [33]. Moreover, Bousarghin et al revealed that HPV-16, -31, and -58, which were closely related viruses, however, presented different endocytosis pathways, HPV-16 and -58 typically internalized through clathrin-coated vesicles, and HPV-31 most likely to involve caveolae [34]. However, one investigation reached a conclusion that HPV-31, as in the case of HPV-16, entered the human and primate cells through a clathrin-mediated pathway [35]. All suggests much ambiguity in terms of papillomavirus entry, which merits further studies.

### 2.2 Caveolar endocytosis

The number of viruses that enter cells via caveolar endocytosis as an alternative uptake pathway was found to be less than via clathrin-mediated endocytosis. In the former, most of these viruses were nonenveloped and less than 55 nm [31], as in the case of HPVs. Compared with the clathrin-mediated entry, caveolae performed internalization at a lower speed, the resulting vesicles failing to become acidized, and an additional difference was that internalization via caveolae was not a constitutive process [32]. Other studies showed that the caveolar endocytosis passed through the caveosomes, bypassing endosomes, and then moved to the Golgi body, and/or endoplasmic reticulum (Fig. 1C) [36], and HPV-31 was found to do this via caveolar endocytosis [34,36,37].

For years, clathrin-dependent endocytosis and caveolar entry were believed to be of two parallel but separate pathways. However, as indicated by the latest investigations, there was cross talk whereby cargo could move between them with some molecules involved. Rab 5 GTPase was first identified. Laniosz et al found that BPV-1 although shown to possess the entry capacity via clathrin-dependent endocytosis, was incapable of establishing an infection without caveolin-1, suggesting that the virus whose entry was facilitated via clathrin-mediated endocytosis, utilized the caveolar pathway postentry for infection, where the Rab 5 might induce or be involved in its transport from the endosome to the caveosome (Fig. 1D) [38]. Afterwards, Smith et al reported that HPV-31, upon initial cellular binding and associating with caveolin-1, was transferred to the early endosome and proceeded through the endosomal pathway, during which the Rab 5 might be responsible for the exchange of the cargo [38]. Another putative molecule might be dynamin, also called a GTPase, which was capable of affecting "pinching off" coated vesicles to form nascent clathrin-coated or caveolin-1-coated endocytic vesicles at the plasma membrane. It was reported that a dynamin inhibitor, dynasore, blocked the infection of HPV-16 and BPV-1 pseudovirions in a dose- and time-dependent manner with equal efficiency [39], and that HPV-31 infection could be blocked using a dynamin-2 dominant negative molecule [35].

### 2.3 A Clathrin- and caveolae-independent pathway

In a latest study, HPV-16 was reported to be capable of entering and thus infecting cells in a clathrin- and caveolae-independent manner, and further evidence indicated that tetraspanin-enriched microdomains (TEMs) were involved in the endocytosis (Fig. 1F) [40]. Tetraspanins are an evolutionary conserved family of four transmembrane domain-containing proteins including at least 32 members in humans [41], which are able to interact laterally with each other and with other transmembrane proteins to form TEMs, within which tetraspanins can control and modulate complicated activities including adhesion, migration, and synapse formation, as well as endocytosis and exocytosis [42]. Spoden et al proposed that HPV-16 particles, following binding to the cells, colocalized with the tetraspanins CD63 and CD151 whose capacity to interact with other membrane components and assemble into microdomains on the plasma membrane enabled these molecules to serve as the recipients of virions from the primary receptors, such as HSPGs. Consequently, the binding could trigger endocytic uptake processes and infection [40].

## 3. L2 protein necessary for infection

The function of L2 has long been neglected. Recently, a growing body of evidence has suggested that L2 is necessary for the establishment of HPV infection. L2 of all sequenced HPVs contain at their N termini a consensus cleavage motif for furin, a proprotein convertase, and furin cleavage is supposed to be necessary for cellular attachment and entry. Therefore, a model of L2 functioning in the early events of PV infection was proposed, in which the initial attachment to HSPGs moieties functioned primarily as the critical step of L2 cleavage by furin, thus resulting in a conformational change of viral capsids, followed by the capsids detaching from HSPGs and associating with a putative second receptor [43]. Other studies showed that furin cleavage might occur at the cell surface or within an early endosomal compartment [44], and the capsids underwent uncoating in a late endosomal compartment, leading to the associated genome to escape from the endosome into the cytoplasm via a mechanism that involved the C-terminus of L2 [45].

In summary, HPVs had to undergo a complicated process to successfully infect their host cells. We presented cellular receptor-binding and internalization pathways of HPVs, which were of multiple steps relating to numerous molecules, cellular or viral, suggesting that it was a promising step in attacking pathogenic viruses before they could utilize the host cell's machinery for replication, and the studies on HPV cellular binding and entry would locate novel molecular targets for antiviral strategies.

## Competing interests

The authors declare that they have no competing interests.

## Authors' contributions

Both authors contributed to the original drafts of the manuscript, and approved the final version.
